# Supplementary material for: Expression of NK Cell Receptor Ligands on Leukemic Cells Is Associated with the Outcome of Childhood Acute Leukemia
Source: Cancers (Basel). 2021 May 11;13(10):2294. doi: 10.3390/cancers13102294 (PMC8151902; doi:10.3390/cancers13102294)
Supplement: Supplementary file 1 [file cancers-13-02294-s001.zip › cancers-1175385-supplementary.pdf]

Table S1. HLA-I allele frequencies in controls and patients.

HLA-allotype	**Controls** (n = 83)	**Patients** (n = 102)	**B-ALL** (n = 70)	**T-ALL** (n = 16)	**AML** (n = 16)
**HLA-A** *01	20 (24.1 %)	21 (21.6 %)	16 (24.6 %)	3 (18.8 %)	2 (12.5 %)
*02	29 (34.9 %)	37 (38.1 %)	29 (44.6 %)	4 (25.0 %)	4 (25.0 %)
*03	12 (14.5 %)	19 (19.6 %)	11 (16.9 %)	4 (25.0 %)	4 (25.0 %)
*11	11 (13.3 %)	14 (14.4 %)	12 (18.5 %)	0 (0 %)	2 (12.5 %)
*23	5 (6.0 %)	7 (7.2 %)	5 (7.7 %)	0 (0 %)	2 (12.5 %)
*24	18 (21.7 %)	21 (21.6 %)	14 (21.5 %)	4 (25.0 %)	3 (18.8 %)
*25	1 (1.2 %)	2 (2.1 %)	1 (1.5 %)	1 (6.3 %)	0 (0 %)
*26	9 (10.8 %)	8 (8.2 %)	7 (10.8 %)	0 (0 %)	1 (6.3 %)
*29	11 (13.3 %)	12 (12.4 %)	7 (10.8 %)	4 (25.0 %)	1 (6.3 %)
*30	13 (15.7 %)	11 (11.3 %)	5 (7.7 %)	2 (12.5 %)	4 (25.0 %)
*31	2 (2.4 %)	3 (3.1 %)	2 (3.1 %)	1 (6.3 %)	0 (0 %)
*32	5 (6.0 %)	5 (5.2 %)	3 (4.6 %)	0 (0 %)	2 (12.5 %)
*33	4 (4.8 %)	5 (5.2 %)	2 (3.1 %)	3 (18.8 %)	0 (0 %)
*68	12 (14.5 %)	11 (11.3 %)	6 (9.2 %)	1 (6.3 %)	4 (25.0 %)
**HLA-B** *07	9 (10.8 %)	11 (11.3 %)	6 (9.2 %)	3 (18.8 %)	2 (12.5 %)
*08	9 (10.8 %)	12 (12.4 %)	8 (12.3 %)	3 (18.8 %)	1 (6.3 %)
*14	8 (9.6 %)	11 (11.3 %)	7 (10.8 %)	3 (18.8 %)	1 (6.3 %)
*15	7 (8.4 %)	10 (10.3 %)	7 (10.8 %)	1 (6.3 %)	2 (12.5 %)
*18	17 (20.5 %)	12 (12.4 %)	8 (12.3 %)	1 (6.3 %)	3 (18.8 %)
*27	3 (3.6 %)	1 (1.0 %)	1 (1.5 %)	0 (0 %)	0 (0 %)
*35	**10 (12.0 %)**	**33 (34.0 %)**[1]	23 (35.4 %)	4 (25.0 %)	6 (37.5 %)
*38	6 (7.2 %)	10 (10.3 %)	7 (10.8 %)	2 (12.5 %)	1 (6.3 %)
*39	5 (6.0 %)	10 (10.3 %)	7 (10.8 %)	1 (6.3 %)	2 (12.5 %)
*40	10 (12.0 %)	9 (9.3 %)	5 (7.7 %)	2 (12.5 %)	2 (12.5 %)
*44	19 (22.9 %)	19 (19.6 %)	14 (21.5 %)	4 (25.0 %)	1 (6.3 %)
*49	11 (13.3 %)	5 (5.2 %)	5 (7.7 %)	0 (0 %)	0 (0 %)
*50	5 (6.0 %)	7 (7.2 %)	5 (7.7 %)	1 (6.3 %)	1 (6.3 %)
*51	15 (18.1 %)	7 (7.2 %)	5 (7.7 %)	2 (12.5 %)	0 (0 %)
*52	0 (0 %)	4 (4.1 %)	3 (4.6 %)	0 (0 %)	1 (6.3 %)
*53	3 (3.6 %)	6 (6.2 %)	2 (3.1 %)	1 (6.3 %)	3 (18.8 %)
*57	5 (6.0 %)	5 (5.2 %)	3 (4.6 %)	2 (12.5 %)	0 (0 %)
*58	4 (4.8 %)	0 (0 %)	0 (0 %)	0 (0 %)	0 (0 %)
**HLA-C***01	2 (2.4 %)	9 (8.8 %)	7 (10.0 %)	0 (0 %)	2 (12.5 %)
*02	7 (8.4 %)	6 (5.9 %)	3 (4.3 %)	1 (6.3 %)	2 (12.5 %)
*03	9 (10.8 %)	11 (10.8 %)	8 (11.4 %)	1 (6.3 %)	2 (12.5 %)
*04	**17 (20.5 %)**	**34 (33.3 %)**[2]	22 (31.4 %)	4 (25.0 %)	8 (50.0 %)
*05	20 (24.1 %)	20 (19.6 %)	15 (21.4 %)	2 (12.5 %)	3 (18.8 %)
*06	10 (12.0 %)	16 (15.7 %)	10 (14.3 %)	3 (18.8 %)	3 (18.8 %)
*07	35 (42.2 %)	34 (33.3 %)	25 (35.7 %)	6 (37.5 %)	3 (18.8 %)
*08	8 (9.6 %)	13 (12.7 %)	9 (12.9 %)	3 (18.8 %)	1 (6.3 %)
*12	11 (13.3 %)	23 (22.5 %)	17 (24.3 %)	4 (25.0 %)	2 (12.5 %)
*14	3 (3.6 %)	1 (1.0 %)	1 (1.4 %)	0 (0 %)	0 (0 %)
*15	10 (12.0 %)	7 (6.9 %)	3 (4.3 %)	1 (6.3 %)	3 (18.8 %)
*16	12 (14.5 %)	10 (9.8 %)	5 (7.1 %)	4 (25.0 %)	1 (6.3 %)
*17	6 (7.2 %)	1 (1.0 %)	1 (1.4 %)	0 (0 %)	0 (0 %)

[1] All patients vs. Controls, p=0.0005, Pc=0.009; [1] All patients vs. Controls, p=0.037, Pc=0.185.

Supplementary Figure S1

A

	HLA-B*35 (P=0.0004)	
	No	Yes
Controls	73 (87.9%)	10 (12.0%)
Patients	64 (66.0%)	33 (34.0%)

	HLA-C*04 (P=0.037)	
	No	Yes
Controls	66 (79.5%)	17 (20.5%)
Patients	68 (66.7%)	34 (33.3%)

C

Controls	HLA-C*04 (P<0.0001)	
HLA-B*35	No	Yes
No	65 (89.0%)	8 (11.0%)
Yes	1 (10.0%)	9 (90.0%)

Patients	HLA-C*04 (P<0.0001)	
HLA-B*35	No	Yes
No	56 (87.5%)	8 (12.5%)
Yes	8 (24.2%)	25 (75.8%)

B

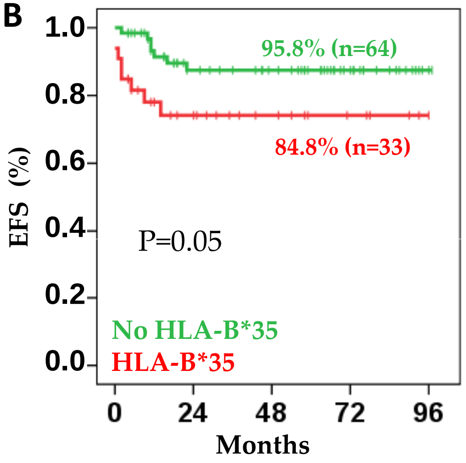

D

Cox regresion for EFS

Co-variable	HR	95%CI	P
Sex	1.270	0.408-3.961	0.680
Age	1.002	0.991-1.013	0.667
Leukemia type	0.912	0.486-1.748	0.803
**Risk status**	**2.751**	1.208-6.268	**0.016**
ULBP1	3.883	0.730-20.65	0.112
**KIR2DL1/C*04**	**4.239**	1.073-16.75	**0.039**
HLA-B*35	1.148	0.306-4.308	0.837

HR. hazard ratio; 95%CI. 95% confidence interval

Figure S1. Reduced event-free survival (EFS) of acute leukemia patients associated with HLA-B*35 is due to its linkage disequilibrium with HLA-C*04. (a) Chi-squared test for HLA-B*35 or HLA-C*04 in controls and patients. Both HLA-B*35 and HLA-C*04 are significantly associated with acute leukemia. (b) Kaplan-Meier and Log-Rank tests for EFS of acute leukemia patients according to the presence of HLA-B*35. HLA-B*35 is associated with reduced EFS. (c) Chi-squared test for HLA-B*35 and HLA-C*04 in controls and patients. HLA-B*35 is in linkage disequilibrium with HLA-C*04 both in controls and in patients. KIR2DL1/HLA-C*04 interaction results in reduced EFS in HLA-B*35 patients. (d) Cox regression analysis for sex, age, leukemia type, ULBP expression on leukemic cells, KIR2DL1/HLA-C*04 interaction and HLA-B*35 allotype. KIR2DL1/HLA-C*04 interaction and risk-status are independent prognostic factors, whereas HLA-B*35 is not.

Supplementary Figure S2

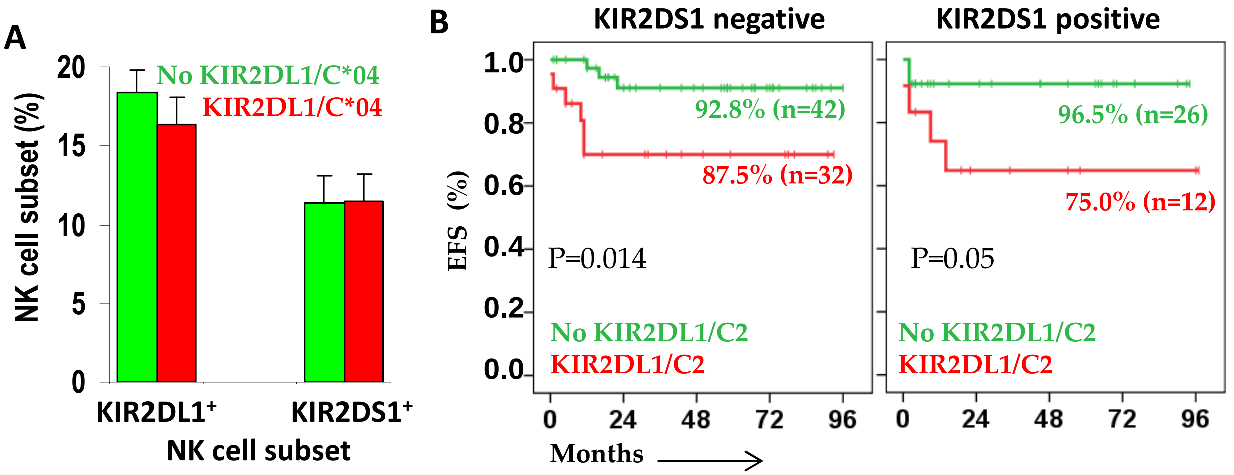

C

	B	ET	Wald	gl	Sig.	Exp(B)	95,0% IC para Exp(B)	
							Inferior	Superior
Sex	,266	,582	,209	1	,648	1,305	,417	4,082
Age	,003	,006	,268	1	,605	1,003	,992	1,014
Leukemia_type	-,146	,338	,185	1	,667	,864	,446	1,677
Standard_Risk	1,050	,423	6,147	1	,013	2,857	1,246	6,551
ULBP	1,385	,851	2,651	1	,104	3,996	,754	21,179
KIR2DL1_C04	1,552	,560	7,673	1	,006	4,723	1,575	14,167
KIR2DS1	,317	,602	,277	1	,599	1,373	,422	4,471

Figure S2. KIR2DS1 did not alter the event-free survival (EFS) curves of acute leukemia pediatric patients according to the presence of KIR2DL1/HLA-C*04 interaction. (a) Frequency of NK cells expressing KIR receptors for HLA C2-epitope (inhibitory KIR2DL1 and activating KIR2DS1) in the peripheral blood of pediatric acute leukemia patients according to the absence or presence of the KIR2DL1/HLA-C*04 interaction. (b) Kaplan-Meier and Log-Rank tests for EFS according to the presence of KIR2DL1/C*04 interaction in KIR2DS1 negative and positive patients. (c) Cox regression analysis for sex, age, leukemia type, ULBP expression on leukemic cells, KIR2DL1/HLA-C*04 interaction and presence of KIR2DS1 gene. The presence of KIR2DS1 gene did not alter the EFS curves according to the presence of KIR2DL1/HLA-C*04 interaction.

Table-S2. Allogeneic stem cell transplantation performed in our series.

							Receptor HLA-ligands				Donor KIR and HLA-ligands										D->R alloreactivity models[1]	
Patient Id	Type leukemia	Cause of Inclusion	Relapse	Death	OS months	HSCT Type	A3 A11	Bw4 ligand	C ligand	HLA-C*04	KIR 2DL1	KIR 2DL2	KIR 2DL3	KIR 3DL1	KIR 3DL2	KIR 2DS1	A3 A11	Bw4 ligand	C ligand	HLA-C*04	Receptor ligand	Ligand incompatibility
p050	LMA	**Relapse**	**Yes**	**Yes**	11	Haplo	No	No	C1C2	**Yes**	1	0	1	**1**	**1**	0	No	Yes	C2C2	**Yes**	3DL1 & 3DL2	3DL1
p070	LMA	**Relapse**	**Yes**	**Yes**	29	Haplo	Yes	No	C2C2	**Yes**	1	**1**	**1**	**1**	1	0	Yes	No	C2C2	**Yes**	2DL2/L3 & 3DL1	None
p014	LLA-B	**Relapse**	**No**	**Yes**	1	UD	Yes	No	C1C2	**Yes**	1	1	1	**1**	1	0	Yes	No	C1C2	**Yes**	3DL1	None
p040	LLA-B	**Relapse**	**No**	**Yes**	11	UD	No	Yes	C2C2	**Yes**	1	0	**1**	1	**1**	0	No	Yes	C2C2	**Yes**	2DL3 & 3DL2	None
p056	LLA-B	**Relapse**	No	No	50	Haplo	No	No	C1C2	**Yes**	1	1	1	0	**1**	**1**	No	No	C1C2	**Yes**	3DL2 & 2DS1	2DS1
p002[2]	LMA	New diagnosis	**Yes**	No	26	UD	No	No	C1C2	No	1	1	1	**1**	**1**	**1**	No	No	C1C2	No	3DL1/L2 & 2DS1	2DS1
			**Yes**	**Yes**		Haplo					1	0	1	**1**	**1**	0	No	Yes	C1C2	No	3DL1 & 3DL2	3DL1
p007	LMA	New diagnosis	**Yes**	**Yes**	14	RD	Yes	No	C2C2	**Yes**	1	0	**1**	**1**	1	1	Yes	No	C2C2	**Yes**	2DL3 & 3DL1	None
p017	LLA-B	New diagnosis	**Yes**	No	86	Haplo	No	No	C1C2	No	1	0	1	**1**	**1**	1	No	No	C2C2	No	3DL1 & 3DL2	None
p030	LLA-T	New diagnosis	**Yes**	No	75	RD	No	No	C2C2	**Yes**	1	0	**1**	**1**	**1**	1	No	Yes	C2C2	**Yes**	2DL3 & 3DL1/L2	3DL1
p006	LMA	New diagnosis	No	No	94	UD	Yes	Yes	C1C2	No	1	0	1	1	1	0	Yes	Yes	C1C2	No	None	None
p009	LLA-T	New diagnosis	No	No	93	UD	No	Yes	C1C2	No	1	1	1	1	**1**	**1**	No	Yes	C1C2	No	3DL2 & 2DS1	2DS1
p010	LLA-B	New diagnosis	No	No	92	RD	No	Yes	C1C2	No	1	0	1	1	**1**	0	No	Yes	C1C2	No	3DL2	None
p049	LMA	New diagnosis	No	No	58	UD	No	No	C2C2	**Yes**	1	**1**	**1**	**1**	**1**	0	No	Yes	C2C2	**Yes**	2DL2/L3 & 3DL1/L2	3DL1
p063	LLA-B	New diagnosis	No	No	43	RD	Yes	No	C1C2	**Yes**	1	1	1	**1**	1	0	Yes	No	C1C2	**Yes**	3DL1	None
p068	LLA-T	New diagnosis	No	No	32	RD	No	Yes	C1C2	**Yes**	1	1	1	1	**1**	**1**	No	Yes	C1C2	**Yes**	3DL2 & 2DS1	2DS1
p078	LLA-B	New diagnosis	No	No	21	UD	No	No	C1C2	No	1	1	1	**1**	**1**	0	No	Yes	C1C2	No	3DL1 & 3DL2	3DL1
p084	LLA-B	New diagnosis	No	No	17	Haplo	No	No	C1C2	**Yes**	1	1	1	**1**	**1**	0	No	Yes	C1C1	No	3DL1 & 3DL2	3DL1
p086	LMA	New diagnosis	No	No	15	UD	No	No	C1C1	No	**1**	0	1	**1**	**1**	1	No	No	C1C1	No	2DL1 & 3DL1/L2	None

HSCT: stem cell transplantation; Haplo: haploidentical HSCT; RD: related donor; UD: unrelated donor; OS: overall survival.

[1] Symons HJ et cols. Biol Blood Marrow Transplant 2010;16(4):533-42. doi: 10.1016/j.bbmt.2009.11.022.

[2] This patient received UD-HSCT and Haplo-HSCT after relapsing.

Cumulative incidence

**Models**		**Relapse**	**Death**
**Receptor Lig,**	n	%	%
KIR2DL1	1	0%	0%
KIR2DS1	4	25%	0%
KIR3DL2	13	31%	23%
KIR3DL1	13	54%	38%
KIR2DL2/L3	4	50%	50%
**Ligand incompatibility**			
KIR2DS1	4	25%	0%
KIR3DL1	6	50%	33%
None	9	44%	44%
